# Conformation-controlled binding kinetics of antibodies

**DOI:** 10.1038/srep18976

**Published:** 2016-01-12

**Authors:** Marta Galanti, Duccio Fanelli, Francesco Piazza

**Affiliations:** 1 Università degli Studi di Firenze, Dipartimento di Fisica e Astronomia and CSDC, via G. Sansone 1, IT-50019 Sesto Fiorentino, Firenze, Italia; 2 Dipartimento di Sistemi e Informatica, Università di Firenze, Via S. Marta 3, IT-50139 Florence, Italy; 3 INFN, Sezione di Firenze, Italia; 4 Université d’Orléans, Centre de Biophysique Moléculaire, CNRS-UPR4301, Rue C. Sadron, 45071, Orléans, France

## Abstract

Antibodies are large, extremely flexible molecules, whose internal dynamics is certainly key to their astounding ability to bind antigens of all sizes, from small hormones to giant viruses. In this paper, we build a shape-based coarse-grained model of IgG molecules and show that it can be used to generate 3D conformations in agreement with single-molecule Cryo-Electron Tomography data. Furthermore, we elaborate a theoretical model that can be solved exactly to compute the binding rate constant of a small antigen to an IgG in a prescribed 3D conformation. Our model shows that the antigen binding process is tightly related to the internal dynamics of the IgG. Our findings pave the way for further investigation of the subtle connection between the dynamics and the function of large, flexible multi-valent molecular machines.

Unarguably, antibodies can be counted among the most important molecular machines for the functioning of life. Like other large biomolecular assemblies, they are increasingly being exploited in modern nanobiotechnology^1^ and biomedical^2^ applications.

Immunoglobulins G (IgG) are large molecules comprising three grossly ellipsoidal domains of length about 6 nm, two Fab arms (prolate) and an Fc stem (oblate), connected by a flexible hinge^3^,^4^,^5^. The tips of the Fab domain host hypervariable regions^6^. These are often referred to as the *active* sites or *paratopes*, as these are the portions of the structure where antigens are bound (at their *epitopes*). The Fc stem is recognized at its lower end by the complement system^7^ and by phagocytic cells^8^ in the early steps of an immune response.

A key property of IgGs is their extreme flexibility, which allows them to adopt a wide range of conformations. Atomic-force microscopy^9^ and single-molecule cryo-electron microscopy (cryo-EM)^10^ measurements have revealed that Fab-Fab and Fab-Fc angles are virtually limited only by steric clashes, with measured values ranging from 15° to 128° (Fab-Fc angle) and from about 20° to 180° (Fab-Fc angle)^10^.

The intrinsic flexibility of IgG molecules reflects their astonishing ability to bind antigens of different sizes, from small molecules such as hormones to large viruses^11^,^12^. Moreover, high flexibility is also key to double-Fab (bivalent) binding to large viruses^13^, a process quantified by the so-called binding *avidity*^14^,^15^, as opposed to the single-binding affinity. Bivalent binding increases the overall strength of the immune response and also allows for IgG-mediated virion aggregation^16^. Recently, it was demonstrated that the intrinsic flexibility of antibodies can be exploited to have them literally *walking* on antigen-covered surfaces with specific lattice-like arrangements of haptens with lattice spacing matching the IgG *stride*^17^.

In this paper we focussed on the following question. Given their great flexibility, it is interesting to assess whether IgGs are more effective in binding a co-diffusing antigen when adopting certain specific conformations. More generally, it would be extremely informative to establish a quantitative link between the large-scale dynamics of antibodies and their binding *effectiveness*. Here we concentrated on this problem in the case of small antigens, where the dynamics of substrate and IgG molecules are characterized by widely separated time scales. More precisely, our model assumes that the characteristic time scale of relaxation of fluctuations of antigen concentration is much faster than the time scale associated with large-scale conformational rearrangements of IgG molecules. In practice, the antigens should diffuse sufficiently fast so to *see* the antibody virtually frozen in one of the many allowed configurations (counted with their associated weights, as dictated by the dynamics). Working in this framework, we shall elaborate on the role of IgG conformation on antigen-antibody diffusion-limited reaction rate.

In order to tackle the above problem, we first constructed a simple coarse-grained (CG) model of the antibody, where each domain is replaced by a rigid structure made of a collection of hard spheres joined by stiff bonds. This was made so as to preserve the overall three-dimensional *shape* of the domains, which are joined together at the hinge and are free to fluctuate about one another, except for the mutual excluded-volume interactions^10^. Remarkably, we show that this mechanical model is enough to recover the experimental distributions of inter-domain angles. In order to quantify the role of the IgG conformation in the diffusive encounter with small antigens, we then elaborated a theory to compute exactly the rate to capture of a small molecule to one of the active Fab tips for an arbitrary configuration of the CG antibodies.

## Results

**Figure 1:**
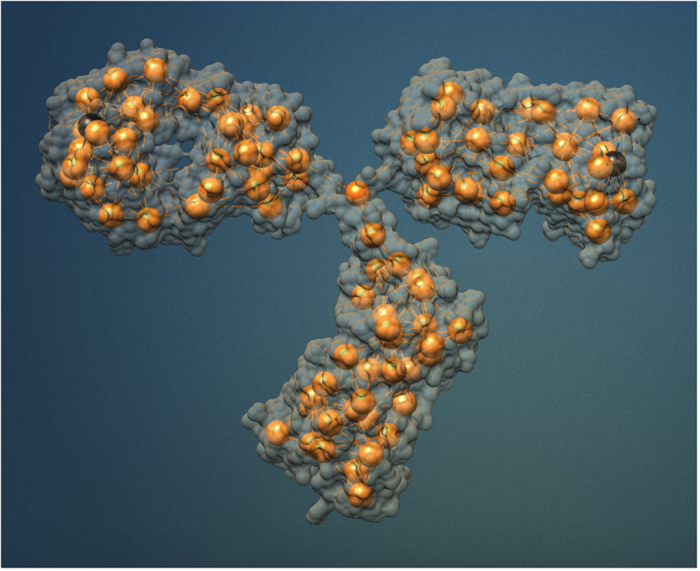
Coarse-grained representation of an IgG with *N* = 96 beads. The *N*-bead structure was obtained by applying the structure-based coarse-grained algorithm implemented in VMD^37^ to the (murine) IgG crystallographic structure with PDB code 1IGT (shown as transparent surface). The dark spheres located at the outer edges of the Fab arms represent the two active sites. The radius of the beads shown in the figure corresponds approximately to the one used in the simulations (0.44 nm, see Methods).

Despite the recent astonishing progresses demonstrated by the massively parallel supercomputer Anton^18^, atomistic molecular dynamics simulations of proteins^19^,^20^ is still an impractical tool for obtaining many conformations of large, flexible molecules such as antibodies. In order to generate many independent configurations of an IgG, we constructed a bead-based CG model (see Fig. 1). In our model, effective beads are joined by stiff springs, that preserve the crystallographic shape of the three domains while they fluctuate about the common hinge region (see Methods).

### Low-resolution, large-scale dynamics of IgGs in solution can be explained by a simple CG model in vacuum

**Figure 2:**
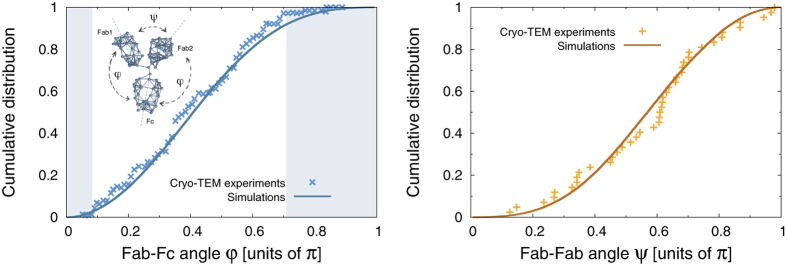
Left panel: cumulative distribution of the Fab-Fc angle *ϕ*. The shaded area identify the regions that are inaccessible because of steric hindrance. Right panel: cumulative distribution of the Fab-Fab angle *ψ*. The solid line stands for the results of our simulations, while symbols refer to the Cryo-Electron Tomography (Cryo-TEM) experiments reported in ref. 10. The inset in the left panel provides a schematic view of the mechanical model employed in the simulation, together with a definition of the relevant angles *ψ* and *ϕ*.

Figure 2 shows the statistics of the Fab-Fab and Fab-Fc angles obtained from a collection of configurations sampled from a constant-energy trajectory of our CG model in vacuum. The striking conclusion is that a simple shape-based coarse-grained model that is able to reproduce the correct shape of the three domains suffices to reproduce the experiments. We stress that in our simulations there is no solvent and the IgG’s domains fluctuate about the hinge (the center of mass is at rest) only subject to mutual collisions, while preserving their shape due to the stiff springs stretched among the beads. The reason behind this somewhat surprising find is that we are looking at equilibrium properties. It is then manifestly redundant that collisions with the solvent add to the random collisions among the domains - they would only generate an *equivalent* noise spectrum that would not change the equilibrium statistics. This also shows that hydrodynamic effects seem not to affect to an appreciable extent the large-scale structural fluctuations of IgGs, which appear mainly controlled by the excluded-volume effects related to the *shape* of the mutually hinged domains.

### An IgG behaves as a nearly isolated dumbbell carrying two binding sites at its ends

Once a plausible coarse-grained dynamical representation of the IgG obtained, we turn now to assessing the role of conformations on the binding rate of small antigens. Antigen-antibody binding can be seen as a two-step chemical reaction of the following type


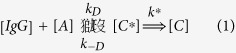


The first step leads to the formation of the encounter complex C^*^. This process is driven by diffusion (rate constant *k*_*D*_). Once the encounter complex is formed, this can be either fixed chemically (with rate constant k^*^), giving rise to a stable antigen-antibody complex or else the two molecules can diffuse away (rate constant *k*_−*D*_). This model assumes that antigen binding is a quasi-irreversible process, *i.e*. the time scale associated with the spontaneous dissociation of the antigen-antibody complex is much longer than the rate of chemical fixation, in line with the high affinity of antigen-antibody binding in general^21^. In the quasi-stationary approximation, valid under conditions of antigen excess where it is safe to set 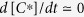, the reaction scheme (1) reduces to


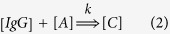


with *k* = *k*_*D*_k^*^/(k^*^ + *k*_−*D*_). In the case of chemical reactions connoted by a great chemical affinity, such as antigen-antibody reactions, one has k^*^ ≫ *k*_−*D*_ and the overall reaction is thus limited by diffusion, with rate constant 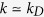. Of course, the rate *k*_*D*_ in eq. (1) should take into account the possibility that the encounter complex be formed with the antigen sitting at either paratope. In the case of a molecule carrying two binding sites at fixed distance this can be simply taken into account through a degeneracy factor of two.

In this paper, we argue that *k*_*D*_ and, more generally, *k* in eq. (2) depend in fact on the actual configuration of the IgG. More precisely, we set out to estimate this effect in the hypothesis that the time scale associated with large-scale conformational changes of an IgG molecules are much longer than the diffusive relaxation time of a small antigen ligand in solution. This can be made more quantitative through the following simple argument. The typical time of large-scale conformational re-arrangements of an IgG molecule can be approximated with the time required for a Fab to explore the space diffusively along an approximately circular path around the Fc. The diffusion constant *D*_*r*_ of a sphere of radius *R*_Fab_ tethered on the surface of a sphere of radius 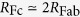 is given by 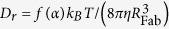, where *f*(*α*) is a geometrical factor that depends on the (inclination) angle *α* formed by the Fab and Fc axes^22^. A figure of merit that quantifies the regime of validity of our model can be defined as the ratio *Q* between the time required for an antigen of size *a*_*L*_ to diffuse across an IgG molecule and the time required for a Fab to diffuse along a semi-circular path on the Fc surface at an average inclination (*α* = 45°). Taking the size of an IgG molecule to be one Fc diameter, *i.e*. approximately 4*F*_Fab_, this gives^22^


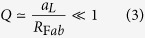


Taking *R*_Fab_ to be of the order of 5 nm^3^,^4^,^5^, we conclude that our theoretical model is valid for *a*_*L*_ ≪ 5 nm.

Let *ρ*_*A*_(**r**, *t*) denote the antigen concentration at time *t* in a fixed laboratory Cartesian frame. As commonly done in the framework of diffusion-influenced reactions^23^, the steady-state (*t* → ∞) rate constant *k* corresponding to a given conformation of the IgG, can be computed by solving the stationary diffusion (Laplace) equation ∇^2^*ρ*_*A*_ = 0 with a suitable set of boundary conditions (BC) and subject to the asymptotic condition 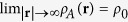, where *ρ*_0_ represents the *bulk* concentration of antigens. The IgG configuration is *hidden* in the boundary conditions, which are imposed on a collection of disconnected spherical boundaries at given locations in space Ω_*α*_ (*α* = 1, ..., *N*) (see Methods). Two of them, the outer spheres at the Fab tips, are considered as perfectly absorbing boundaries (sinks). This amounts to imposing a perfectly absorbing boundary condition of the kind 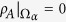, which physically describes a reactive boundary endowed with an infinitely fast chemical reactivity^24^. The *N* − 2 spheres left can be endowed in principle with a specific *intrinsic* reaction rate k^*^. In general, according to the general scheme (1), this could be used to model competitive binding to a more complicated, multi-valent surface. In this paper we shall restrict to k^*^ = 0 for non-paratope regions, which corresponds to making the IgG molecule perfectly reflecting to antigens, except for the perfectly absorbing active spheres located at the Fab tips.

**Figure 3:**
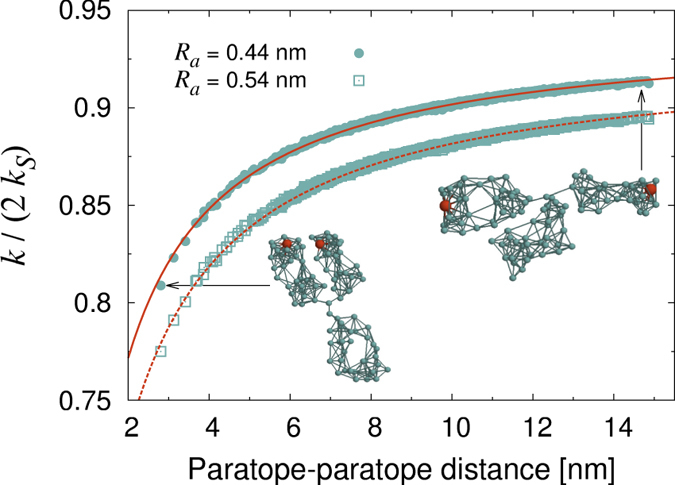
Rate constant as a function of the distance between the active sites (red beads in the snapshots) for two different choices of the encounter distance *R*_*a*_ = paratope size + antigen size. The rate constant is normalized by twice the Smoluchowski rate constant of an isolated paratope, *k*_*S*_ = 4*πDR*_*a*_, where 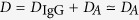 is the relative diffusion constant (practically equal to the antigen diffusion coefficient). Each symbol represents the rate calculated for a given configuration of the IgG. The radius of the reflecting spheres that define the body of the IgG was fixed at 0.44 nm in both cases. The solid and dotted lines refer to the effective model (4) multiplied by a constant factor *f*_*a*_. Here *f*_*a*_ = 0.94 (*R*_*a*_ = 0.54 nm) and *f*_*a*_ = 0.93 (*R*_*a*_ = 0.44 nm). Other parameters are *N* = 96.

The above posed boundary problem can be solved analytically for a given configuration of the IgG to any desired accuracy (see Methods). In Fig. 3, we plot the rate constant *k* as a function of the distance between the two binding sites (Fab tips). As a first observation, it can be recognized that the rate constant is always smaller than twice that of equivalent but isolated paratope spheres. This reduction stems from the competition between the two paratopes for the target antigen molecule^25^,^26^ and from non-trivial screening effects exerted by the reflecting IgG body^27^. Remarkably, the points displayed in Fig. 3 fall on a smooth curve. The absence of appreciable scattering around the average profile suggests that the relative distance between the paratopes is a meaningful parameter that is well suited to provide a quantitative characterization of the binding performance of a dynamical IgG. The reflecting beads of the IgG body shield out part of the incoming flux of antigens. However, such screening seems not to depend on how the inert spheres are arranged around the paratopes, so long as these are at a fixed distance.

The results reported in Fig. 3 can be interpreted by resorting to an effective and rather intuitive model. The IgG can be replaced by a dumbbell made of two spherical sinks of radius *R*_*a*_ placed at a distance *d*. The rate constant for such a configuration can be computed analytically to any desired accuracy^23^. In the monopole approximation one has


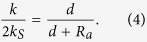


where *k*_*S*_ = 4*πD*_*A*_*R*_*a*_ is the rate constant of an isolated paratope (see caption of Fig. 3). We note that the paratope *size R*_*a*_ is the *encounter* distance, *i.e*. the sum of the linear size of the active site and that of the antigen. Figure 3 clearly shows that eq. (4) can be used to reproduce the data provided it is multiplied by a constant reduction factor *f*_*a*_ < 1, namely *k*/(2*k*_*S*_) = *f*_*a*_*d*/(*d* + *R*_*a*_). This factor is the only remnant of the IgG body (the other *N* − 2 reflecting spheres), whose action on the two sinks only causes a rather modest 6 ÷ 7% reduction with respect to an isolated paratope dumbbell.

**Figure 4:**
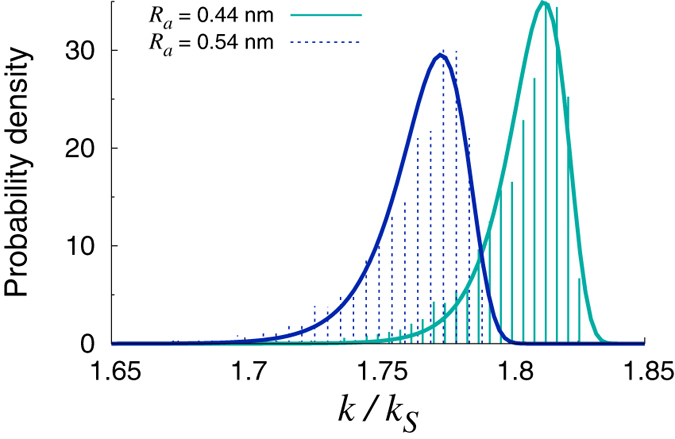
Histograms of the reaction rate constants computed for different choices of *R*_*a*_. The solid lines are fits to the Gumbel density distribution 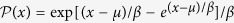, where *μ*, *β* are the fitting parameters. The best-fit values of the parameters are: *μ* = 1.81, *β* = 0.010 (*R*_*a*_ = 0.44 nm), *μ* = 1.77, *β* = 0.012 (*R*_*a*_ = 0.54 nm).

To shed further light on the above findings, it is instructive to look at the distributions of the computed reaction rate constants. These are shown in Fig. 4. One can appreciate how the flexibility of the IgG impacts the statistics of the rate constants. The profiles appear negatively skewed, which implies that the internal dynamics of the IgG molecule favors on average those configurations which are associated with larger values of the reaction rates. Interestingly, the histograms can be fitted to Gumbel profiles, which suggests that the rate constants computed from the sampled configurations may correspond to near-maximum sampled values. Moreover, the curves shift towards the right, namely to higher values of the normalized rate constants, when *R*_*a*_ is reduced. This is a consequence of the reduced diffusion interaction between the two active sites. In fact, the larger the active regions, the greater the rate constant for an isolated paratope, but the smaller the combined rate constants for the two-paratope system^23^,^25^,^26^.

### Going ultra-coarse-grained: the three-sphere IgG

**Figure 5:**
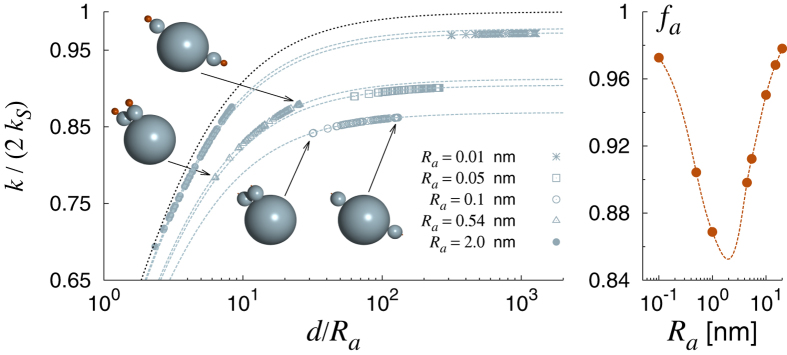
Left: rate constant normalized by 2*k*_*s*_ versus rescaled paratope-paratope distance for different choices of the paratope size *R*_*a*_. The grey dashed lines are plots of formula (4), rescaled by appropriate normalization factors *f*_*a*_ as shown in the right panel (the solid line is only a guide to the eye). The black dotted line is the plot of the perfect dumbbell prediction, *k*/2*k*_*S*_ = *f*_*a*_[*d*/(*d* + *R*_*a*_)] with *f*_*a*_ = 1.

The results illustrated so far hold in general, and do not depend on the level of coarse-graining imposed in building the effective bead-based model of IgGs. The same analysis can be repeated for different choices of *N*, yielding similar conclusions. However, one could in principle speculate that a sphere-based model will inevitably yield a structure with plenty of *holes*, unphysical consequences of the coarse-graining procedure. The antigens, assumed point-like as their size is incorporated in the size of the paratope beads, *R*_*a*_, could diffuse through the structure and this could be the reason why the whole IgGs behaves quite just like a nearly perfect dumbbell. In order to investigate further into this matter, we studied a hyper-simplified model where the IgG is replaced by three contacting spheres, two representing the Fab arms and one modeling the Fc stem. The active paratopes were still modeled as two additional absorbing spheres at the outer ends of the Fab spheres (see cartoons in Fig. 5).

Figure 5 illustrates our results for this ultra-coarse-grained model. Overall, the picture traced in the previous paragraph is confirmed, which shows that the partially transparent nature of the multi-bead model does not introduce artifacts in the computation of the rate constant and can be safely adopted as it allows one to sample the large-scale configuration space in agreement with the experiments. The rate constant in the three-bead model can still be described by a modified (rescaled) dumbbell: the largest rate constants are invariably associated with the configurations where the Fab arms are stretched away from each other to a maximum, while the smallest rate constants correspond unfailingly to the situation where the active sites are close to each other (see cartoons in Fig. 5). The rescaling factor to be used in the dumbbell law is close to one for very large and very small sizes of the paratopes. In the former case, the reason is that the active spheres are strong enough not to feel the screening of the three-bead reflecting IgG. In the latter case, the paratopes are so small that their separation is always several times their size. At intermediate sizes of the active sites the reduction is largest.

## Discussion and Summary

In this paper we have developed a coarse-grained dynamical model of IgG antibodies, where each domain is described by an almost rigid, shape-preserving arrangement of hard spheres joined by stiff springs. The three domains are free to rotate about the common hinge. Constant-energy simulations in vacuum reproduced with good accuracy the statistic of the main structural parameters, as extracted from single molecule Cryo-Electron Tomography (Cryo-ET) experiments, with no adjustable parameters. We conclude that the equilibrium distribution of large-scale coordinate of IgGs and possibly, by the same token, of other large flexible biomolecule is essentially determined by excluded-volume constraints. In order to capture the true equilibrium properties, these have to be taken into account by a faithful description of the different *shapes* of the relevant domains. This is the first non trivial find of this work.

In the following part of the paper, we have developed an exact theory to compute the rate constant for the formation of an antigen-antibody complex for an arbitrary configuration of the IgG. We have found that the IgG behaves as a nearly isolated dumbbell-like molecule carrying two active paratope spheres at its ends. The relevant structural parameter that determines the rate constant is therefore the paratope-paratope distance. The rate constant in this model can be computed analytically through a simple formula. Furthermore, a careful analysis of the rate constant distributions over the population of sampled IgG conformations revealed that the higher weight is associated with larger values of the rate constant. Therefore, we conclude that internal flexibility forces the antibody to visit preferentially those configurations that are associated with higher probability of forming an encounter complex.

Summarizing, our results suggest that large, flexible molecules may have been designed by evolution to exploit their flexibility to a maximum degree in terms of the ability of binding small antigens. Of course, this is only part of the story. IgGs bind also bivalently to different epitopes on the same surface, such as a virus capsid. Thus, the mutual flexibility and shape of the three lobes must also have been shaped by the evolutive pressure exerted by requiring such binding events to be optimized.

Unfortunately, it seems rather hard to design an experiment with the aim of assessing the role of different conformations of antibodies on the antigen binding rate. Conventional techniques to perform kinetics measurements, such as Surface Plasmon Resonance (SPR)^28^ or fluorescence-based methods^29^, only return *average* measures over the whole conformational ensemble. One solution could be to compare measurements performed with the same antigens on wild-type antibodies and on specifically designed molecules, whose conformational ensembles are confined to selected portions of their phase space. This might be the case of antibodies designed to partially reduce or, even delete the hinge domain, so inducing a substantial loss of segmental flexibility^30^. An alternative route could be to compare the kinetics of (i) individual antigen-binding fragments (Fabs) and (ii) assemblies of two, three or more recombined such fragments with the kinetics of intact antibodies^31^. Another interesting and viable route to test the predictions of our theory would be to analyze antibodies from camelids, such as dromedary or llamas^32^,^33^. About half of the antibodies of these animals lack light chains and only feature two heavy chains with three IgG domains each^33^, with the missing IgG domain replaced by a flexible linker. The IgG domain carrying the complementarity determining regions (CDR) display two point mutations that make their surface more hydrophilic (where normal IgG feature the interface between the terminal light-chain and heavy-chain CDR-carrying domains). This has prompted researchers to isolate these special IgG domains, which are now known under the name of *nanobodies* and hold great promise for the biotechnology industry^32^. Interestingly, nanobodies show the same high affinities of full antibodies and can be used to construct more complex molecules, such as by joining two of them via a long and flexible hinge. It would be interesting to compare kinetics measurements performed on a host of different antigen-binding systems, such as the one described above, to validate our theoretical approach.

Concluding, we have introduced a novel theoretical framework adapted to study the role of structural fluctuations of large biomolecules in the formation of complexes with small ligands. The results discussed in the framework of antigen-antibody reactions are encouraging and point to a tight and subtle relation between the ability of biological macromolecules to form complexes and their internal dynamics. Our method is utterly general and can be applied in many contexts. For example, to investigate the role of large-scale structural fluctuations of DNA in the formation of DNA-protein complexes or to examine the role of enzyme conformations in the formation of enzyme substrate complexes. As single-molecule cryo-EM is rapidly emerging as a powerful technique for determining the structure of flexible biomolecules at high resolution^34^, our approach might represent an invaluable tool in the new era of structural/dynamical biology.

## Methods

### Coarse-grained model of IgGs

The coarse-grained model of antibodies was built with the shape-based algorithm^35^,^36^ implemented in the VMD package^37^, by choosing *N* = 96 spheres. This number is a good trade-off between the constraints of keeping the structure light enough (fewer spheres) and reproducing faithfully enough the three-dimensional *shape* of the domains (more spheres). The diameter of the beads composing the IgG was set equal to the smallest center-to-center distance found in the CG assembly, multiplied by 0.95 (to prevent tangency of contiguous beads). Based on this criterion, we obtain *R*_*α*_ = 0.44 nm. The resulting assembly of spheres was then bound into a scaffolded structure by requiring all spheres within a cutoff radius *R*_*c*_ = 22 Å within each other to be joined by very stiff springs (stiffness *κ* = 50 kcal/mol/Å^2^). This *de facto* prevents the domains to *breathe* excessively and only allows the three domains to rotate freely around one another about the common hinging point (see again Fig. 1). The steric repulsion was taken into account through a potential energy of the form 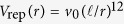 with *v*_0_ = 5 kcal/mol and 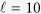 Å. The total potential energy of our model reads then


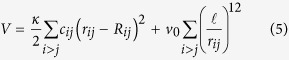


Here the position of the *i*-th bead as a function of time and in the equilibrium structure are denoted by **r**_*i*_(*t*) and **R**_*i*_, respectively. Accordingly, *R*_*ij*_ = |**R**_*j*_ − **R**_*i*_| and *r*_*ij*_(*t*) = |**r**_*j*_(*t*) − **r**_*i*_(*t*)| are the equilibrium and displaced inter-bead distances, respectively. The matrix *c*_*ij*_ = (1 if *R*_*ij*_ < *R*_*c*_|0 otherwise) specifies all the interacting pairs, and is known as the connectivity matrix.

A set of different configurations was then obtained by sampling a constant-energy trajectory with initial condition given by the crystallographic structure (PDB code: 1IGT). In order to reconstruct the trajectory, we integrated Newton’s equation numerically.


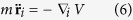


through a position-extended Forest-Ruth like (PEFRL) symplectic algorithm^38^ with a time step *dt* = 2.8 fsec. The mass *m* of the effective beads was fixed at 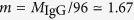 kDa.

The trajectory was sampled every 0.56 psec to obtain the coordinates of different conformers. The Fab-Fab and Fab-Fc angles were obtained for each conformer by first constructing the inertial ellipsoids of the three domains. The three angles (*ψ* and the equivalent *ϕ*_1_, *ϕ*_2_) were then computed from the scalar products of the vectors describing the major axes of the three ellipsoids (pointing outward from the hinge).

### Calculation of the binding rate constant

The stationary normalized density *u*(**r**) = *ρ*_*A*_(**r**)/*ρ*_*B*_ is a solution of the Laplace equation with boundary conditions imposed on the surfaces of the spheres Ω_*α*_ of radius *R*_*α*_ (*α* = 1, 2,…, *N*) that compose the IgG molecule (see Fig. 1). The boundary problem to be solved can be posed in a rather general form as follows,


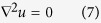



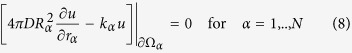



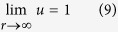


where *k*_*α*_ is the intrinsic rate constant of the *α*-th spherical boundary. BCs of the kind (8) are called *radiative* and account in general for an intrinsic reactivity of the boundaries according to the kinetic scheme (1)^39^. Let *α* = 1, 2 denote the two spheres at the tip of the Fab arms (the paratopes, black spheres in Fig. 1). Here we take


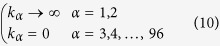


Accordingly, BCs in eq. (8) become perfectly absorbing, namely 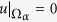, for the paratopes, while the rest of the IgG structure is perfectly reflecting. Once the Laplace equation is solved (see below for details), the binding rate constant can be computed as the total flux into the active sites of the IgG, namely


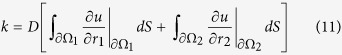


#### Solving the Laplace equation

In order to solve the Laplace equation in the assigned manifold, we introduce as many sets of spherical coordinate systems as there are spherical boundaries. The solution can then be written formally as an expansion in series of irregular solid harmonics, namely


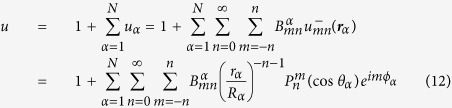


where 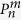 are associated Legendre functions and *r*_*α*_ the radial coordinate associated with the spherical system which is centered on the *α*-th sphere. The coefficients 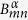 should be determined by imposing the boundary conditions (8). In order to do so, we can use known addition theorems for the spherical harmonics^40^ to express the solution (12) in all the *N* different reference frames centered at each sphere. As a result, we obtain an infinite-dimensional system of linear equations for the unknown coefficients 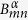



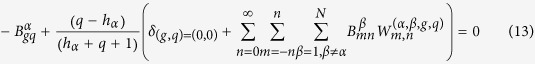


for all *α* = 1, 2, …, *N*, *q* = 1, 2, ... ∞ and *g* = −*q*, ..., *q*, where *h*_*α*_ = *k*_*α*_/(4*πDR*_*α*_). Our choice (10) corresponds to *h*_*α*_ → ∞ for *α* = 1, 2 and *h*_*α*_ = 0 for *α* ≠ 1, 2. However, we remark that our solution is more general and can be employed in principle to investigate binding to more complicated, multi-valent surfaces. The matrix elements of *W* read


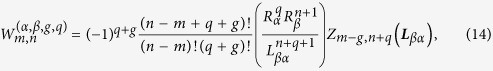


where ***L***_*βα*_ is the vector which connects the centers of Ω_*β*_ and Ω_*α*_ in the direction Ω_*β*_ → Ω_*α*_ and 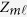 is defined as


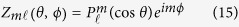


To solve the above system one needs to truncate the sum on *n* in eq. (13), by including a finite number of multipoles, *N*_*t*_, which should be chosen so as to attain the desired accuracy. Recalling the definition (11), the ligand binding rate constant *k* can be computed explicitly. This gives


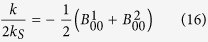


where *k*_*S*_ = 4*πDR*_*a*_ is the Smoluchowski rate constant for an isolated paratope of encounter radius *R*_*a*_ (= radius of the active site + radius of the ligand).

## Additional Information

**How to cite this article**: Galanti, M. *et al*. Conformation-controlled binding kinetics of antibodies. *Sci. Rep*. **6**, 18976; doi: 10.1038/srep18976 (2016).

## Supplementary Material

Supplementary Information
