# Supplementary material for: Conformation-controlled binding kinetics of antibodies
Source: Sci Rep. 2016 Jan 12;6:18976. doi: 10.1038/srep18976 (PMC4709514; doi:10.1038/srep18976)
Supplement: Supplementary Information [file srep18976-s1.pdf]

# Supplemental information to the paper "Conformation-controlled binding kinetics of antibodies", by Marta Galanti, Duccio Fanelli and Francesco Piazza

## Clarification of the motivation for the kinetic model in Eq (1)

Eq. (1) represents the simplest kinetic scheme to describe the binding of a ligand to an IgG in solution. First an encounter complex is formed, which can either dissociate as the two reactants (IgG and antigen) diffuse away from one another, or become fixed as the ligand binds tightly to one of the IgG paratopes. Furthermore, this model assumes that antigen binding is a quasi-irreversible process, *i.e.* the time scale associated with the spontaneous dissociation of the antigen-antibody complex is much longer than the rate of chemical fixation, in line with the high affinity of antigen-antibody binding in general [Kumagai2001].

Of course, the rate $k_D$ in eq. (1) should take into account the possibility that the encounter complex be formed with the antigen sitting at either paratope. In the case of a molecule carrying two binding sites at fixed distance this can be simply taken into account through a degeneracy factor of two. We argue that $k_D$ (and, more generally, $k$ in eq. (2)) depend in fact on the actual configuration of the IgG. Our paper sets out to estimate this effect in the hypothesis that the time scale associated with large-scale conformational changes of an IgG molecules are much longer than the diffusive relaxation time of a small antigen ligand in solution.

More complex kinetic models involving multi-valent antigens (necessarily larger than what assumed in our paper) at an interface are well known in the literature (see *e.g.* the studies reported in Refs. [Sadana1997] and [Yang2003]). However, these kinetic schemes, besides being adapted to large antigens with multiple binding sites, assume that one of the reactants, either the antibody or the antigen, is immobilized on a surface, as it happens on surface-plasmon resonance experiments or in antibody-based biosensors.

## Conditions of validity of Eq. (2)

Eq. (2) holds under quasi-stationary conditions (see for example [Houston2001]). The rate equations corresponding to the kinetic scheme (1) read

$$\begin{cases} \dfrac{d[C^*]}{dt} = k_D[IgG][A] - (k_{-D} + k^*)[C^*] & \text{(i)} \\ \dfrac{d[C]}{dt} = k^*[C^*] & \text{(ii)} \end{cases}$$

Under conditions of ligand excess, one can assume that a quasi-stationary state is quickly reached, where the encounter complex concentration [C*] stays practically constant. Letting d[C*]/dt = 0 in eq. (i) above, one can compute [C*] as a function of the antibody and antigen concentrations, namely

$$[C^*] = \frac{k_D}{k^* + k_{-D}}[IgG][A] \qquad \text{(iii)}$$

Substituting expression (iii) in eq. (ii) and comparing with the rate equation corresponding to the kinetic model (2) in the main text, one immediately obtains the explicit expression for the effective rate $k$ cited in the paper (immediately below eq. (2)).

## A critical discussion of the assumption of frozen IgG conformations

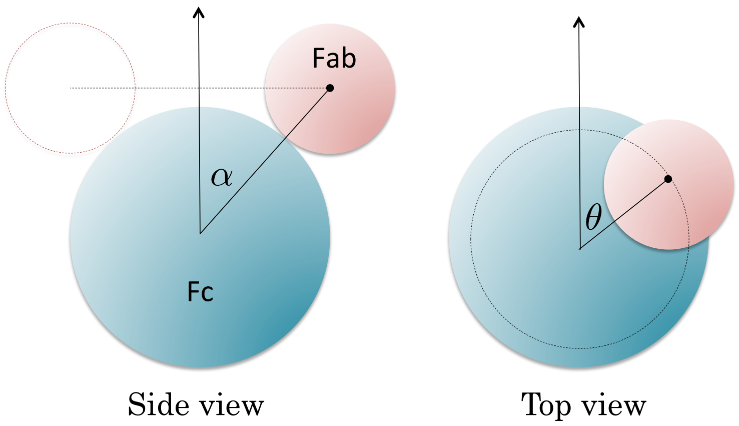


$$\langle \theta^2 \rangle = 2D_r(\alpha)t$$

where

$$D_r(\alpha) = f(\alpha)\frac{k_B T}{8\pi\eta R_{\mathrm{Fab}}^3}$$

**Figure 1**. Schematic representation of the circular diffusional motion of a Fab arm bound to the Fc stem in an IgG (broken circle). The diffusion coefficient is proportional to the free rotational diffusion coefficient, with a proportionality constant that decreases with the Fab inclination $\alpha$ [Iniesta1987].

Our model assumes that the antigens diffuse sufficiently fast with respect to the typical time of large-scale conformational rearrangements of an IgG molecule. This problem might at first sight be attacked from the perspective of gated reactions (see for example [McCammon 2011]). However we find it a highly non-trivial task to model typical large-scale conformational dynamics as a single gating rate. In order to provide an estimate of the maximum antigen size for which our theoretical treatment holds, we can instead proceed as follows.

We can model a Fab as a sphere of radius $R_{\mathrm{Fab}}$ tethered on the surface of a greater sphere, representing the Fc stem of the antibody molecule. Due to the great flexibility of the Fab-Fc link and to the large size of the Fab domain, we can assume that the typical time for such a Fab sphere to diffuse in a circular path on the Fc surface should provide a correct order of magnitude for the typical time scale of large-scale conformational re-arrangements that we are seeking (see Fig. 1). We observe that such conformational dynamics is expected to be diffusive, due to the high flexibility of the hinge linker. Unfortunately, we cannot use our molecular dynamics scheme to extract the sought for time scale, as our simulations are conducted in vacuum. Therefore, we can employ our framework only to provide a correct sampling of the conformational ensemble (as it is shown in the paper by comparing to cryo-ET experiments). However, we cannot rely on the associated dynamics to estimate realistic time scales.

With reference to Fig. 1, the diffusion constant of a sphere tethered by a flexible linker on the surface of another sphere is given by [Iniesta1987],

$$D_r(\alpha) = f(\alpha)\frac{k_B T}{8\pi\eta R_{\mathrm{Fab}}^3}$$

where the reduction factor $f < 1$ depends on the inclination of the Fab with respect to the Fc axis (see Fig. 1). Taking the IgG to be in an average configuration at an inclination of about 45° yields $f = 0.25$ [Iniesta1987]. The figure of merit that quantifies the regime of validity of our model can be defined as the ratio $Q$ between the time required for an antigen to diffuse across an IgG molecule and the time required for a Fab to diffuse along a semi-

circular path on the Fc surface. Taking the $R_{\mathrm{Fc}} = 2R_{\mathrm{Fab}}$ and taking the size of an IgG molecule to be one Fc diameter we get

$$Q \simeq \frac{a_L}{R_{\mathrm{Fab}}}$$

where $a_L$ is the radius of the ligand (antigen) molecule. Hence, we conclude that our theory is sound for antigens such that $Q << 1$, which translates approximately to $a_L << 5$ nm, if we approximate one Fab with a sphere of radius $R_{\mathrm{Fab}} = 5$ nm.

## References


[Desmyter2015] Desmyter, A., Spinelli, S., Roussel, A., & Cambillau, C. (2015). Camelid nanobodies: killing two birds with one stone. Current Opinion in Structural Biology, 32C, 1–8.

[Houston2001] Houston, Paul L., Chemical Kinetics and Reaction Dynamics (Dover Publications Inc., Mineola, New York, 2001).

[Iniesta1987] Iniesta, A. (1987). Rotational diffusion coefficients of a small, spherical subunit flexibly tethered to a larger sphere. *European Biophysics Journal*, *14*(8), 493–498.

[Kumagai2001] Kumagai, Izumi, and Tsumoto, Kouhei (Apr 2010) Antigen–Antibody Binding. In: eLS. John Wiley & Sons Ltd, Chichester.

[McCammon 2011] McCammon, J. A. (2011). Gated Diffusion-controlled Reactions. BMC Biophysics, 4(1), 4.

[Sadana1997] Sadana, A., & Vo-Dinh, T. (1997). Antibody-antigen binding kinetics a model for multivalency antibodies for large antigen systems. Applied Biochemistry and Biotechnology, 67(1-2), 1–22.

[Spouge1996] Spouge, J. L., Szabo, A., & Weiss, G. H. (1996). Single-particle survival in gated trapping. Physical Review E, 54(3), 2248–2255.

[Yang2003] Yang, T., Baryshnikova, O. K., Mao, H., Holden, M. A., & Cremer, P. S. (2003). Investigations of bivalent antibody binding on fluid-supported phospholipid membranes: The effect of hapten density. Journal of the American Chemical Society, 125(16), 4779–4784.

[Zhou2004] Zhou, H.-X. & Szabo, A. (2004). Enhancement of association rates by nonspecific binding to dna and cell membranes. Phys. Rev. Lett. 93, 178101.
